# Instantaneous Phase Coherence Imaging for Near-Field Defects by Ultrasonic Phased Array Inspection

**DOI:** 10.3390/s20030775

**Published:** 2020-01-31

**Authors:** Haiyan Zhang, Lingtian Zeng, Guopeng Fan, Hui Zhang, Qi Zhu, Wenfa Zhu

**Affiliations:** 1 School Institute for Advanced Communication and Data Science, School of Communication and Information Engineering, Shanghai University, Shanghai 200444, China; authentictseng@126.com (L.Z.); phdfanry@shu.edu.cn (G.F.); zh6154@126.com (H.Z.); 2 School of Urban Railway Transportation, Shanghai University of Engineering Science, Shanghai 201620, China; 3 School of Mechatronic and Automation Engineering, Shanghai University, Shanghai 200444, China

**Keywords:** ultrasonic phased array, instantaneous phase, diffuse field, weighting factor

## Abstract

This paper describes an imaging method for near-field defect detection in aluminum plates based on Green’s function recovery and application of instantaneous phase coherence weighting factors. The directly acquired acoustic information of near-field defects is usually obscured by the nonlinear effects due to the physical limitation of the acquisition system. Using the diffuse field to recover the Green’s function can effectively retrieve the early time information. However, averaging operations of finite number in this process produces an imperfect imaging result. In order to improve the image quality, two kinds of instantaneous phased coherence weighting factors are used to weight the Green’s function to reduce the background noise and improve the signal-to-noise ratio: the instantaneous phase coherence factor (IPCF), and the instantaneous phase weighting factor (IPWF). Experiments are conducted on two aluminum plates with two and four near-field defects, respectively. As a result, the background noise of amplitude images weighted by IPCF and IPWF is less than that of the conventional total focusing method (TFM). In addition, the IPCF image achieves a better signal-to-noise ratio (SNR) than that of IPWF, and the phase discontinuity in an IPWF image is suppressed through the IPCF.

## 1. Introduction

Metal materials are widely used in plate-like structures. These structures are required to be sufficiently durable and safe in harsh and complicated environments [1,2,3]. Due to factors such as aging and fatigue, damage gradually accumulates in the structure. Such damage can cause serious accidents if it is not detected and repaired in time. Ultrasonic techniques are widely used to detect defects. By emitting high frequency acoustic waves into the material and then analyzing the reflection echoes, defect information can be obtained. Holmes first introduced the concept of full matrix capture (FMC) [4,5], and proposed that the total focusing method (TFM) imaging algorithm be performed on the FMC dataset. By comparing the detection performance of TFM with traditional scanning techniques such as plane B-scan, focused B-scan, and sector scan, it was concluded that TFM provided the most precise information about defects. The FMC dataset from a phased array is the collection of A-scans composed of transmit-receive element pairs, and the TFM is based on the delay and sum (DAS) of signal amplitudes [6,7]. Imaging quality can be improved by synthesizing the phase information in the signal.

The instantaneous phase carries important information hidden in the signal [8]; it can be used as a coherence factor to obtain more precise imaging results. Camacho analyzed the phase information in the acquired signal and proposed application of the phase coherence factor (PCF) and the sign coherence factor (SCF) [9,10]. Camacho’s results showed that PCF can improve the resolution and contrast of ultrasound images. Based on analyses of SCF, Prado proposed a compounding method [11] that used A0 and S0 mode Lamb waves to detect artificial defects in an aluminum plate. These two sets of detected signals were combined in an image with amplitudes weighted by SCF. The experiment preserved the best response of each mode, resulting in reduction of the background noise. Furthermore, Higuti analyzed the instantaneous phase and determined the numerical value of the instantaneous phase threshold [12,13,14] according to the statistical analysis of noise. When the image pixel intensity was above the instantaneous phase threshold, it was judged to be a reflector. If the image pixel intensity was below the instantaneous phase threshold, it could be judged as an artifact. This approach improved reflector detectability and reduced the intensity of image artifacts. However, this instantaneous phase analysis divides the signal into defect and noise signal only by reference to the instantaneous phase threshold value. When the instantaneous phase values change around the defect, the phase discontinuity between the pixels will appear in the imaging result.

In conventional ultrasonic testing, such as the TFM method, there will be a noisy area under the probe, also known as a dead zone, caused by the physical limitations of the acquisition system. The early time acoustic information is obscured, preventing detection of the defect. To solve this problem, Potter et al. [15] proposed calculating the cross-correlation between the diffuse fields in order to retrieve Green’s function, from which the early time information can be reconstructed. The proposed method improves the detection ability of TFM in the near-field range. However, unnecessary noise [16] is generated at the same time, because only a finite number of averaging operations are applied on the diffuse field, which affects the defect identification.

In this paper, the IPCF is proposed to reduce artifacts and improve contrast. This method can effectively reduce the background noise in the cross-correlation process, and its application in the near-field defect detection is investigated. Near-field defects in two aluminum plates were explored by using two factors: the instantaneous phase weighting factor (IPWF) and the instantaneous phased coherence factor (IPCF). The remainder of this paper is organized as follows: In Section 2, the Green’s function retrieval theory and the imaging methods are introduced. Section 3 presents the experimental apparatus and procedures for near-field defect detection. Section 4 shows the experimental results of acquired data processed with conventional TFM, IPWF and IPCF. Finally, the conclusions are given in Section 5.

## 2. Instantaneous Phased Coherence Imaging

### 2.1. FMC and Amplitude TFM

**Figure 1:**
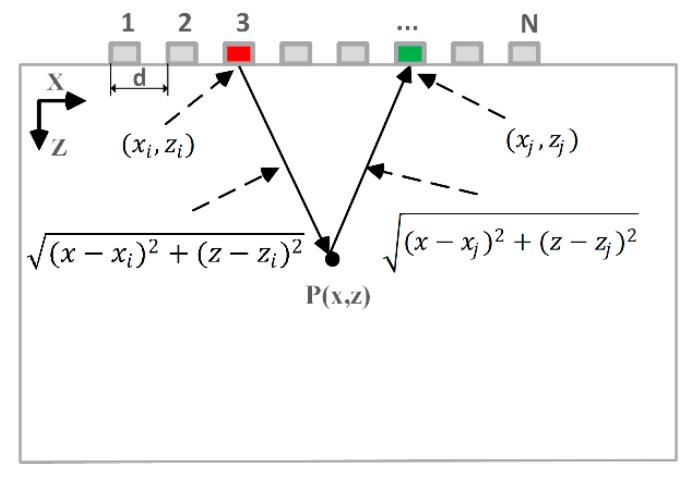
Linear array with N elements, pitch d, and coordinate system.

The amplitude imaging method used in this paper is based on TFM, which utilizes the FMC data to focus at every point in the target region, as shown in Figure 1.

A linear array consisting of *N* elements excites the ultrasonic wave in sequence. During each excitation, the ultrasonic signal is received by every array element. Finally, an N×N A-scans matrix is obtained. Here, *h_ij_*(*t*) is the A-scans signal emitted by element *i* and received by element *j*. The amplitude intensity at a point P(x,z) is calculated by Equation (1) [4]:

(1) $$I_{a}(x,z)=\frac{1}{N^{2}}\sum_{i=1}^{N}\sum_{j=1}^{N}h_{ij}(t_{ij}(x,z))$$

where $t_{ij}(x,z)$ is the time of flight starting from the transmitter *i*, passing through the focusing point P(x,z), and arriving at the receiver *j*. The time of flight is defined by Equation (2):

(2) $$t_{ij}(x,z)=\frac{\sqrt{{\left(x-x_{i}\right)}^{2}+{\left(z-z_{i}\right)}^{2}}+\sqrt{{\left(x-x_{j}\right)}^{2}+{\left(z-z_{j}\right)}^{2}}}{c}$$

where *c* is the velocity of ultrasound, $\left(x_{i},z_{i}\right)$ and $\left(x_{j},z_{j}\right)$ are the Cartesian coordinates of the transmitter and the receiver, respectively.

### 2.2. Green’s Function Recovery

There is a serious obscured area under the probe when TFM is applied during direct contact measurement. Although the near-field signal presents only in the obscured region of direct acquisition, it can be recovered by the cross-correlation of diffuse fields. The diffuse field is an approximately uniform sound field obtained by using sufficient time reflections and scattering in a bounded system. It can be denoted by $d_{ij}(t)$, which is excited by element *i* and received in element *j* after a long transmission time $t_{r}$. Previous work showed that the average value of the cross-correlation at two points is equal to the Green’s function between them [17,18]. The reconstructed Green’s function full matrix $r_{ij}\left(t\right)$ can be written in the same way, as follows:

(3) $$r_{ij}(t)=\frac{1}{N}\sum_{s=1}^{N}\int_{t_{r}}^{t_{r}+t_{c}}d_{si}(\tau )d_{sj}(\tau +t)d\tau$$

where *N* is the number of phased array elements, and $t_{c}$ is the time window length. Actually, the near-field information in the reconstructed matrix is more accurate than the information from the traditional directly captured signal, but becomes imperfect in the later time range. Therefore, a hybrid full matrix consisting of the early time information from the reconstruction and the later information from the traditional direct full matrix can be a good choice for imaging. This hybrid full matrix $m_{ij}(t)$ is given by [15]:

(4) $$m_{ij}(t)=\frac{1}{1+e^{-\alpha (t-t_{u})}}h_{ij}(t)+\frac{\sum_{k=1}^{N}\left|h_{kk}(t_{b})\right|}{\sum_{k=1}^{N}\left|r_{kk}(t_{b})\right|}\left(1-\frac{1}{1+e^{-\alpha (t-t_{u})}}\right)r_{ij}(t)$$

where $t_{u}$ is the transition time when the saturation effects have disappeared from the direct full matrix, $t_{b}$ is the time for an echo to reflect from the back wall, and α is the smoothness parameter of transition, which equals $25\times 10^{6}\;s^{-1}$. Replacing the direct full matrix $h_{ij}(t)$ with the hybrid full matrix $m_{ij}(t)$ in Equation (1), the complete image is described as:

(5) $$I_{a}(x,z)=\frac{1}{N^{2}}\sum_{i=1}^{N}\sum_{j=1}^{N}m_{ij}(t_{ij}(x,z))$$

### 2.3. Instantaneous Phase Coherence Factor

Considering the propagation of the acoustic wave, the instantaneous phase information $\varphi_{ij}(t)$ from the hybrid full matrix $m_{ij}(t)$ is obtained by [19]:

(6) $$\varphi_{ij}(t)=\arctan\left(\frac{{\hat{m}}_{ij}(t)}{m_{ij}(t)}\right)$$

where ${\hat{m}}_{ij}(t)$ is the Hilbert transformation of $m_{ij}(t)$. The instantaneous phase image is determined by using the instantaneous phase $\varphi_{ij}(t)$ in Equation (5):

(7) $$I_{\varphi }(x,z)=\frac{1}{N^{2}}\sum_{i=1}^{N}\sum_{j=1}^{N}\varphi_{ij}(t_{ij}(x,z))$$

The instantaneous phase image is then compared pixel by pixel with the instantaneous phase threshold ε. ε is determined by the statistical analysis of noise, as shown in Equation (8) [12]:

(8) $$\varepsilon =\frac{1}{\sqrt{\log_{10}(N^{2})}}$$

IPWF was first presented by Higuti et al. [13] in their use of instantaneous phase imaging. A defect may exist at a given point, when the $I_{\varphi }(x,z)$ is above the instantaneous phase threshold ε at that point; in that case, the IPWF is set to 1. Otherwise, only noise exists and the IPWF is equal to *P*. Considering these two cases, the IPWF at a point can be written as [13]:

(9) $$IPWF(x,z)=\left\{\begin{array}{l} 1\;\;\mathrm{if}\;\left|I_{\varphi }(x,z)\right|\geq \varepsilon \\ P\;\;\mathrm{if}\;\left|I_{\varphi }(x,z)\right|<\varepsilon \end{array}\right\}$$

where *P* (0<P<1) is the probability of wrong detection of a reflector given by [12]:

(10) $$P=\frac{\sigma_{0}^{2}}{N^{2}}\log(N^{2})$$

where $\sigma_{0}^{2}$ is the variance of noise in the instantaneous phase, which is equal to π/3 for a uniform distribution over 2π. If a reflector is not detected, the IPWF is set to *P* at that pixel. As a result, the intensity is reduced and the background noise is suppressed. The amplitude image processed with IPWF is obtained as follows:

(11) $$I_{\mathrm{IPWF}}(x,z)=I_{a}(x,z)\times IPWF(x,z)$$

Since only two IPWF values, 1 and *P*, can be obtained around the pixel in the image according to the instantaneous phase threshold, the problem of value jumping between adjacent pixels arises. When the instantaneous phase values change around the defect, the discontinuity between the pixels causes peak dispersion in the imaging result.

In order to overcome this problem in IPWF imaging, a new weighting factor modified from the coherence factor map is presented here; it utilizes the in-phase and quadrature components of the signal [20]. The IPCF is defined as:

(12) $$IPCF(x,z)=\frac{1}{N^{2}}{\left\{{\left(\sum_{i=1}^{N}\sum_{j=1}^{N}\sin(\varphi_{ij}(t_{ij}(x,z)))\right)}^{2}+{\left(\sum_{i=1}^{N}\sum_{j=1}^{N}\cos(\varphi_{ij}(t_{ij}(x,z)))\right)}^{2}\right\}}^{\sigma }$$

where the exponent σ≥0 is a variable parameter that controls the sensitivity of the weighting factor. If σ = 0, the IPCF is equal to 1 at any point, and no weighting is applied. When σ = 0.5, IPCF is the envelope form of the instantaneous phase. With an increase in σ, the heights of the side lobes are suppressed and the width of the main lobe is reduced. The IPCF is then continuous in value rather than restricted to only two possible values. After multiplying by the amplitude image *I_a_*(*x*,*z*), the final IPCF image is obtained as:

*I*_IPCF_(*x*,*z*) = *I*_*a*_(*x*,*z*) × *IPCF*_(*x*,*z*)_ (13)

## 3. Experiment 

**Figure 2:**
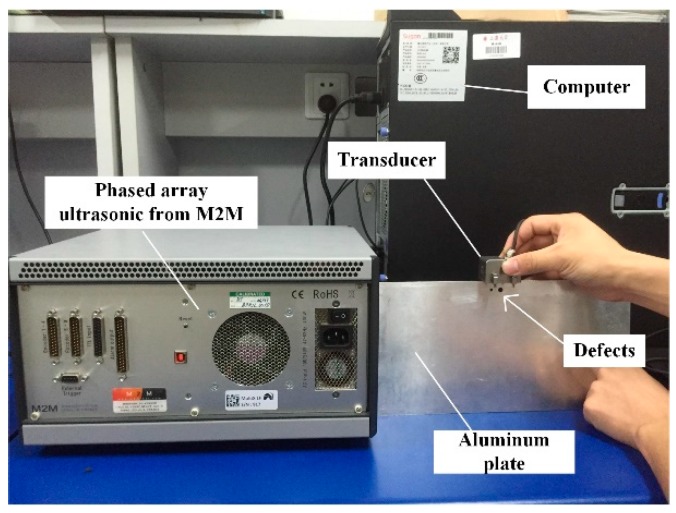
Experimental apparatus.

The experimental system was set up to validate our proposed method. It included a host computer, a commercial phased array controller (Multi2000, M2M Inc, Les Ulis, France), and two thin aluminum plates with defects, as illustrated in Figure 2. The 16-element linear array transducer (Shantou Ultrasonic Electronics Co. LTD) had a central frequency *f* = 1 MHz with an element pitch of 2.0 mm and an element width of 1.8 mm. Each element was excited by a five-cycle sinusoidal signal modulated by a Gaussian window with an excitation voltage of 70 V. Full probe parameters are listed in Table 1.

**Figure 3:**
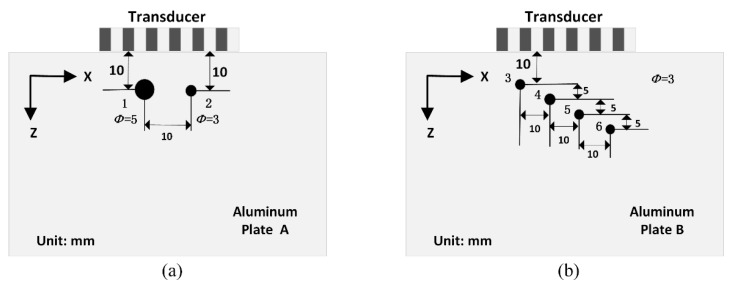
Schematic of defects in: (**a**) aluminum plate A and (**b**) aluminum plate B.

The defect positions in the aluminum plates are shown in Figure 3 in the *x*-*z* plane. The proposed method was tested on two 1-mm-thick isotropic aluminum plates (300 mm ×150 mm). In plate A, there were two through-hole defects (No. 1 and 2) with diameters of 5 mm and 3 mm, respectively, from left to right. The distance between the centers of these two defects was 10mm. Both defects were located 10mm from the upper edge of the aluminum plate. In plate B, there were four through-hole defects (No. 3-6), each with the same diameter of 3 mm. Between each defect, the lateral spacing was 10mm, and the vertical spacing was 5 mm. The distance between defect No. 3 and the upper edge of the aluminum plate was 10 mm.

The Lamb wave has dispersion and multi-mode characteristics which depend on the frequency-thickness product [21]. The transducer can generate many propagation modes of Lamb waves, depending on its geometry, the frequency-thickness product, the orientation of the excitation wave with respect to the plate, and other parameters. The detection capability is related to the mode type and defect geometry. For instance, the S0 mode is sensitive to a defect at any depth, while the A0 mode is more sensitive to surface defects [22]. 

In this experiment, the transducer was positioned at the middle point of the edge of the aluminum plate. An S0 mode Lamb wave was then excited by the method proposed in [23]. The frequency-thickness product in our test was 1 MHz ۰ mm, the S0 mode Lamb wave velocity *v* was approximately 5300 m/s, and the wavelength was λ=v/f=5.3 mm. The near-field distance was $N_{d}=D^{2}/4\lambda$ [24], where *D* was the aperture of the transducer and was equal to 31.8 mm. According to the calculation, $N_{d}\approx 47$ mm; all six defects were located in the near-field range.

**Figure 4:**
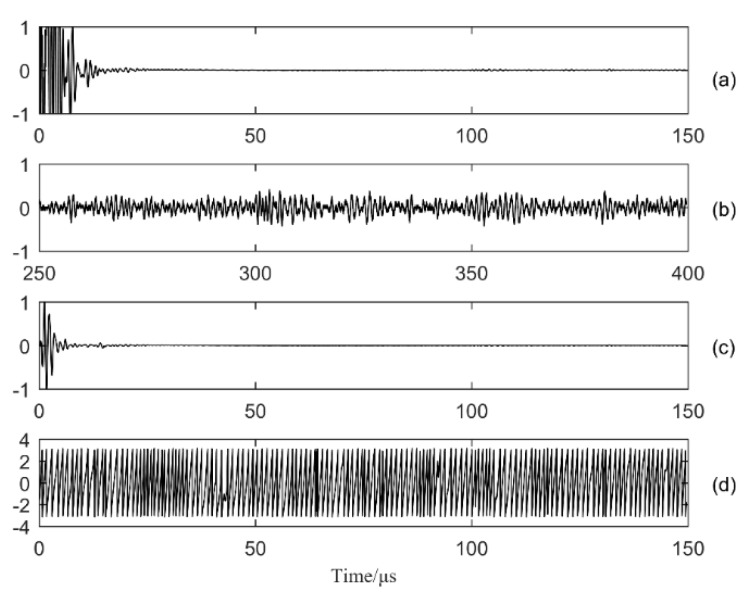
Time traces for i = 16, j = 16 in plate B: (**a**) directly captured signal response; (**b**) diffuse full matrix, $d_{ij}(t)$; (**c)** hybrid full matrix signal, $m_{ij}(t)$; and (**d**) instantaneous phase, $\varphi_{ij}(t)$.

Setting the transducer to full matrix mode, data for a 16 × 16 full matrix were captured using the Multi2000 software. The signals were processed on MATLAB R2016a (MathWorks, Natick, MA, USA). The time window $t_{c}$ was set at 150 μs, and a typical time trace for *i* = *j* = 16 obtained from aluminum plate B is shown in Figure 4. The directly captured signal $h_{ij}(t)$ is shown in Figure 4a, with a noisy area in the early time. The transition time $t_{u}$ is 15 μs, which should be chosen as a time shortly after the saturation effects have disappeared from the direct full matrix. The arrival time $t_{b}$ is 56.6 μs. After $t_{r}\;250\;\mu s$ transmission, the diffuse field is shown in Figure 4b. Figure 4c is the hybrid full matrix $m_{ij}(t)$, composed of Figure 4a,b, and all three signals were normalized to the maximum value. Compared with Figure 4a, the noisy area is significantly reduced in the early time. Figure 4d denotes the instantaneous phase $\varphi_{ij}(t)$ for distribution in the [−π, π] range.

## 4. Experimental Results and Discussion

**Figure 5:**
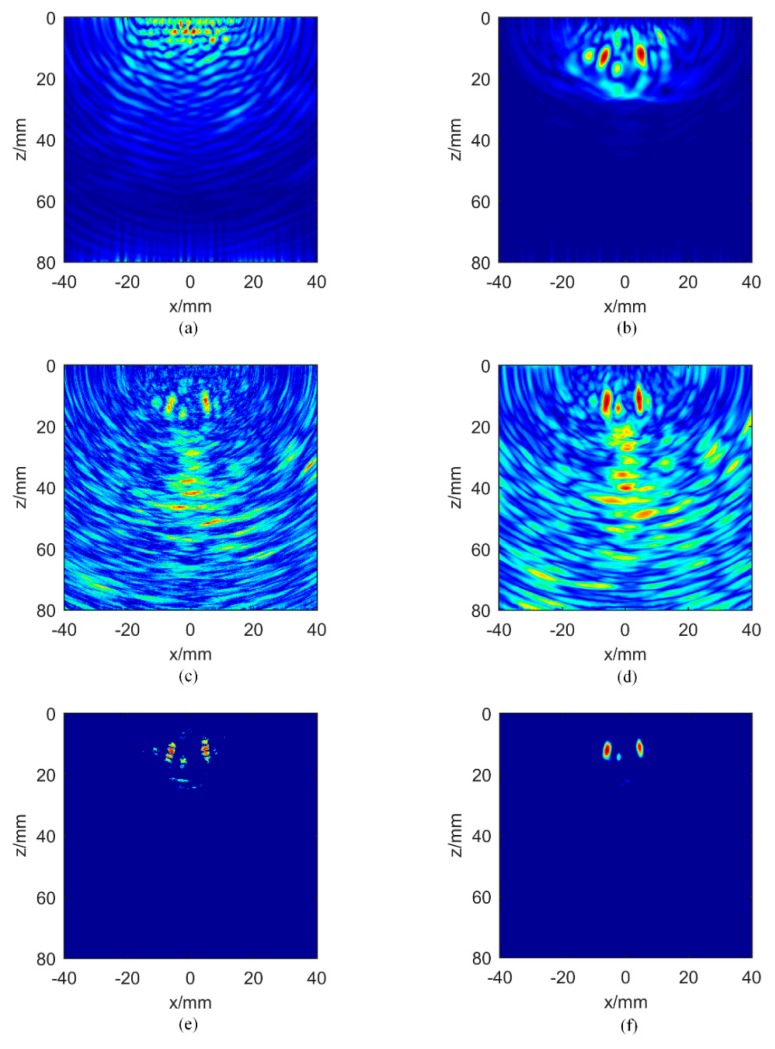
Images of aluminum plate A: amplitude total focusing method (TFM) images for (**a**) the direct full matrix and (**b**) the hybrid full matrix; instantaneous phased coherence weighting factor for (**c**) instantaneous phase weighting factor (IPWF) and (**d**) instantaneous phase coherence factor (IPCF), σ = 1; amplitude images multiplied by (**e**) IPWF and (**f**) IPCF, σ = 1.

Figure 5 presents the imaging results for aluminum plate A. The amplitude TFM images for the direct full matrix $h_{ij}(t)$ and the hybrid full matrix $m_{ij}(t)$ are shown in Figure 5a,b respectively. The early time information of the directly captured signal is obscured by the nonlinear effects of the instrument. The result of direct imaging cannot show the position of the near-field defect correctly. Even though the defects are recovered by using the hybrid full matrix, the finite number of averaging operations in the cross-correlation process also introduces unnecessary noise. Figure 5c is the IPWF obtained by Equation (9), and Figure 5d presents the proposed IPCF with the variable parameter σ = 1. It can be seen that the signal intensity from defects in Figure 5d is higher than that in Figure 5c but with more background noise in the later time information. Figure 5e,f are the amplitude images multiplied by the IPWF and IPCF, respectively. According to Figure 5e, IPWF suppresses the background noise and improves the signal-to-noise ratio. However, since IPWF is not continuous across adjacent pixels, multi-peaks appear in the image. Comparing with Figure 5e, the IPCF image has less background noise, and the phase discontinuity disappears.

**Figure 6:**
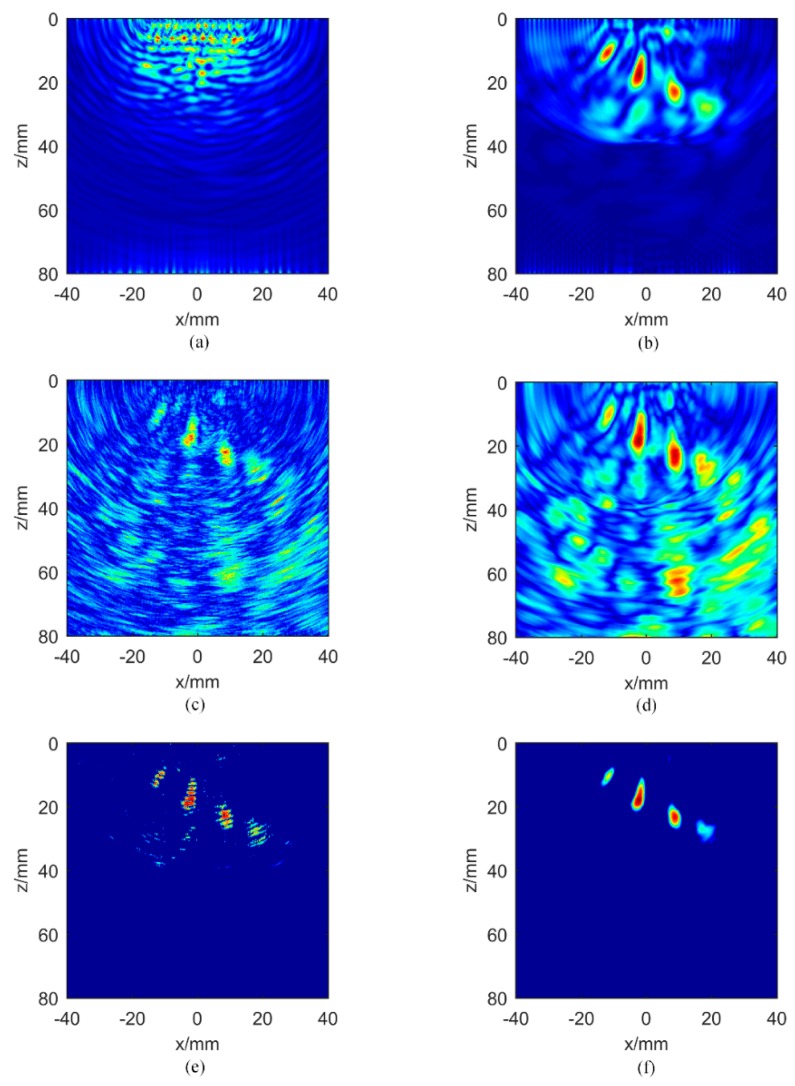
Images of aluminum plate B: amplitude TFM images for (**a**) the direct full matrix and (**b**) the hybrid full matrix; instantaneous phased coherence weighting factor for (**c**) IPWF and (**d**) IPCF, σ = 1; amplitude images multiplied by (**e**) IPWF and (**f**) IPCF, σ = 1.

Figure 6 presents the imaging results of aluminum plate B, which are similar to the case in plate A. The defect in the near field cannot be detected from the directly captured full matrix. The amplitude TFM imaging of the hybrid full matrix $m_{ij}(t)$ is shown in Figure 6b. It can be seen that four defects are detectable, but there are still some artifacts around the defects. The amplitude image multiplied by IPWF is shown in Figure 6e. The background noise is suppressed, but the value near the defect is not continuous. Figure 6f presents the amplitude image multiplied by the IPCF with the parameter σ = 1. Compared to Figure 6b, the background noise in Figure 6f is significantly reduced and the contrast of the image is effectively improved. Figure 6f presents more consistent defect values compared to Figure 6e, but the signal intensity of defect No. 6 begins to decrease. Due to the energy attenuation of the Lamb wave, defect No. 6 is not recovered completely. The experimental results on two aluminum plates show that the IPCF proposed in this paper achieves better imaging results than IPWF in the detection of near-field defects.

**Figure 7:**
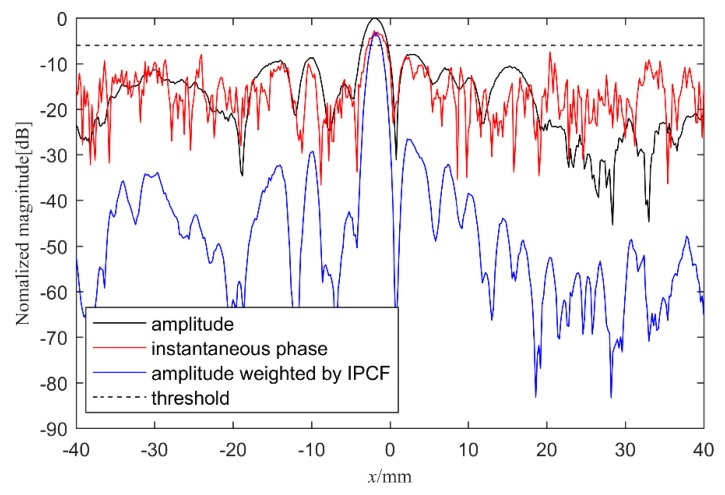
Axial view for *z* = 15 mm in plate B, with amplitude shown with the black line, instantaneous phase shown with the red line, IPCF(σ = 1) shown with the blue line, and instantaneous phase threshold shown with the dashed line.

In order to compare the imaging performance of the three methods, the axial view at z = 15mm in aluminum plate B is shown in Figure 7. All the images are normalized in dB. The amplitude TFM of the hybrid full matrix $m_{ij}(t)$ is represented by the black line, and the instantaneous phase $I_{\varphi }(x,z)$ calculated by Hilbert transform is shown with the red line. It can be seen from Figure 7 that the main lobe height of instantaneous phase and amplitude TFM is similar, with the peak intensity appearing near *x* = 5 mm, which is consistent with the coordinate of defect No. 4. On both sides of the plate, the noise intensity of the instantaneous phase is higher than that from the amplitude TFM. The dashed line indicates the magnitude of the instantaneous phase threshold. By observing the imaging results, it is seen that the instantaneous phase near the main lobe is higher than the threshold, while the instantaneous phase at other places is lower than the threshold. The condition of a signal intensity higher than the threshold is judged as a defect, which is consistent with the actual situation. The amplitude image weighted by IPCF is represented by the blue line. The main lobe value is slightly lower than the amplitude TFM, but the values around the main lobe are lower than the amplitude TFM and instantaneous phase. Therefore, the noise around the defect is significantly suppressed.

For quantitatively analyzing the performance of the three imaging methods, the signal-to-noise ratio (SNR) is used to indicate the relationship between the defect signal and noise. The definition of SNR for a defect can be expressed by [25]:

(14) $$\mathrm{SNR}=20\times \log_{10}(\frac{I_{\max}}{I_{ave}})$$

where $I_{\max}$ is the maximum value of the defect signal in the surrounding region, and $I_{ave}$ is the average value of background noise. Here, we analyzed the SNR of six defects in the two aluminum plates for amplitude TFM, with the amplitude image multiplied by IPWF and IPCF (σ = 1). The results are shown in Table 2. When the amplitude TFM is multiplied by IPWF or IPCF, the SNR is significantly improved. Take defect No. 1 for example: after using the IPWF and IPCF, the SNR is improved by 14.96 dB and 16.47dB, respectively. The SNR for different defects is also improved by using the instantaneous phased coherence weighting factor. The SNR improvements achieved by application of the two instantaneous phased coherence weighting factors are similar. However, the peak values of the IPCF image results are more concentrated compared to the peak values when the IPWF is applied; IPCF thereby produces a better imaging result.

**Figure 8:**
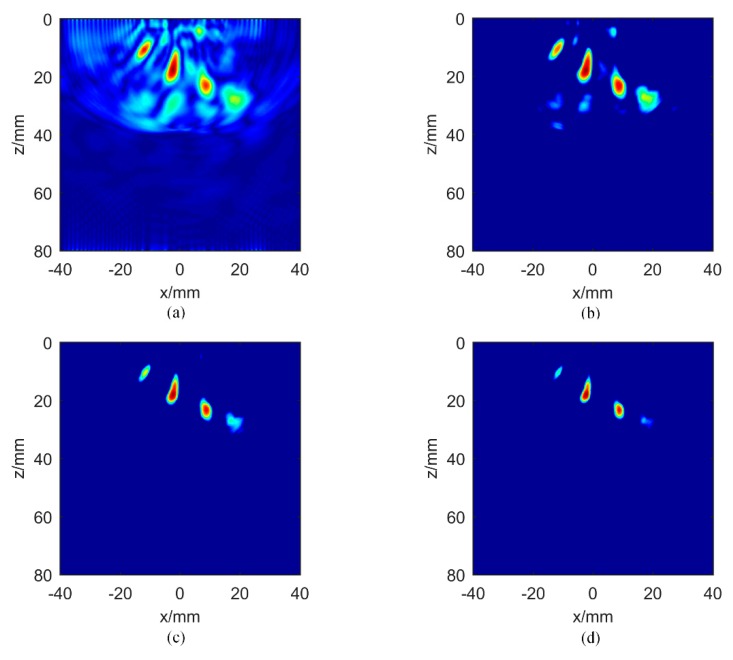
Effect of variable σ on imaging for aluminum plate B: (**a**) σ = 0; (**b**) σ = 0.5; (**c**) σ = 1; (**d**) σ = 1.5.

In order to analyze the influence of the exponent variable σ on IPCF imaging results, four different σ values were tested in aluminum plate B for comparison. The experimental results are shown in Figure 8. When there is no weighting factor, i.e., σ = 0, the IPCF image is equal to the amplitude TFM of the hybrid full matrix $m_{ij}(t)$. When σ = 0.5 (Figure 8b), four defects are detectable, with each defect having high intensity. Noise can be found under defect No. 4 and along the upper edge of the aluminum plate. When σ = 1(Figure 8c), the background noise decreases, and the signal intensity of the four defects is decreased. In particular, it is difficult to detect defect No. 6. When σ is increased to 1.5 (Figure 8d), the signals of defect No. 3 and defect No. 6 begin to disappear. From the above analysis, we draw the following conclusions: When the variable σ increases, the background noise will gradually decrease, and the ability to detect the defect will also be weakened. When the variable σ is reduced, the artifacts may affect the identification of a defect. It should be emphasized that care must be taken when identifying the artifacts and defect signals. By slowly increasing the value of sigma, and comparing the obtained IPWF images with the traditional amplitude TFM image, the intensity of the background noise will decrease rapidly, and the place with high signal intensity and slow noise decline can be considered as the defect location. Therefore, in the process of IPCF imaging, the variable σ should be chosen carefully. Usually, σ = 0.5 or σ = 1 will be a good choice.

## 5. Conclusions

In this paper, IPCF is used to weight the Green’s function for the identification of near-field defects in aluminum plates. It is shown that the proposed weighting factor improves the quality of the amplitude TFM image by reducing the background noise. Compared with IPWF imaging, it retains the advantages of suppressing background noise and overcomes the phase discontinuity. The near-field information can be recovered by using the cross-correlation of the diffuse full matrix, but some unnecessary noise is also introduced at the same time. Applying the IPCF to near-field defect detection improves recovery of the defect information. During the experiment, the M2M ultrasonic phased array was used to excite the S0 mode Lamb waves on two isotropic aluminum plates with through-hole defects in the near-field range. Three different imaging methods are compared. The experimental results show that IPCF imaging can effectively reduce background noise, improve imaging contrast, and improve the damage detection ability. Finally, the influence of the variable σ on the imaging results is analyzed. σ should be carefully selected; in general, 0.5 or 1 is used.

The use of IPCF for near-field imaging provides a means to improve the near-field defect detection capability. However, there remain some limitations of this method, and when IPCF is used, additional calculations are required. Similar to other conventional imaging methods, this method is not sensitive to the shape characteristics of defects. Since the method is based on the information processing and optimal algorithm, there is no theoretical obstacle in applying the method to anisotropic or other materials. In future work, more effective phase weighting factors will be explored for different materials and more complex structures.

## Figures and Tables

**Table 1:** Experimental array parameters.

Parameter	Value
Number of elements	16
Element width	1.8 mm
Element pitch	2.0 mm
Sampling frequency	50 MHz
Center frequency	1 MHz
Excitation voltage	70 V
Wave velocity	5300 m/s

**Table 2:** Signal-to-noise ratio (SNR) of the hybrid full matrix for amplitude TFM (**I**), multiplied by IPWF (**II**) and IPCF (**III)**.

SNR (dB)	Defect No.					
	1	2	3	4	5	6
**Case I**	11.68	9.72	9.63	9.33	8.74	6.97
**Case II**	26.64	25.32	21.34	24.31	24.93	19.21
**Case III**	28.15	27.79	22.02	25.06	26.65	20.97
